# Analysis of Health Benefits Conferred by *Lactobacillus* Species from Kefir

**DOI:** 10.3390/nu11061252

**Published:** 2019-06-01

**Authors:** Conor Slattery, Paul D. Cotter, Paul W. O’Toole

**Affiliations:** 1 School of Microbiology and APC Microbiome Institute, University College Cork, T12 Y337 Cork, Ireland; conorslattery1994@gmail.com; 2 Food Research Centre, Moorepark, Fermoy, Co., P61 C996 Cork, Ireland; paul.cotter@teagasc.ie

**Keywords:** kefir, Lactobacillus, kefiri, kefiranofaciens, plantarum, probiotic

## Abstract

Lactobacilli are among the most common microorganisms found in kefir; a traditional fermented milk beverage produced locally in many locations around the world. Kefir has been associated with a wide range of purported health benefits; such as antimicrobial activity; cholesterol metabolism; immunomodulation; anti-oxidative effects; anti-diabetic effects; anti-allergenic effects; and tumor suppression. This review critically examines and assesses these claimed benefits and mechanisms with regard to particular Lactobacillus species and/or strains that have been derived from kefir; as well as detailing further potential avenues for experimentation.

## 1. Introduction

Kefir is a putative health-promoting probiotic drink produced through the fermentation of milk with kefir grains, which are composed of several fungal and bacterial species. Consumption of kefir has been associated with numerous health benefits, including lower levels of inflammation, anti-carcinogenic effects, lower serum cholesterol levels, improved digestion and gut health, a reduction in hypertension, and regulation of reactive oxygen species. *Lactobacillus* species form a significant part of kefir grains and constitute an overwhelming majority of bacterial species present [1]. While kefir originated in the Caucasus region of Eastern Europe, it has spread worldwide, with artisanal strains being found in Asia, Africa, South America, and Western and Eastern Europe. In this review, we analyze the health benefits associated with the most commonly studied strains of *Lactobacillus* found in kefir.

An initial search of the literature focusing on kefir and the associated *Lactobacillus* species was undertaken. Once the species level analysis was completed, the literature was further subdivided according to the individual strains within these species, and the results presented in Table 1. The most commonly found strains among the literature were then reviewed in greater depth and their purported health benefits summarized. The purported health benefits of less studied strains were also analyzed. This analysis focuses on *Lactobacillus* strains found in *milk* kefir, as opposed to other types of kefir.

The phylogeny of the *Lactobacillus* species found in kefir is diverse. Based on the recent paper by Salvetti et al. relating to *Lactobacillus* taxonomy, *Lactobacillus* species in kefir occupy a variety of subgroups [2], with at least one strain being present in the *Lactobacillus plantarum*, *Lactobacillus delbrueckii*, *Lactobacillus casei*, *Lactobacillus reuteri* and *Lactobacillus buchneri* groups, with other strains being found amongst the *Leuconostoc* and *Lactococcus* genera. However, a significant majority of the kefir-derived *Lactobacillus* strains belonged to the *Lb. delbrueckii* and *Lb. buchneri* groups, mainly because of the presence of *Lactobacillus kefiranofaciens* and *Lactobacillus kefiri* species within these respective groups. These individual species account for a significant proportion of the *Lactobacillus* species found in kefir. *Lb. plantarum* are also known to be present in significant proportions in kefir and have been extensively studied.

## 2. Results

### 2.1. Lactobacillus Plantarum

#### 2.1.1. *Lactobacillus Plantarum* CIDCA 83114

The kefir strain *Lb. plantarum* CIDCA 83114 was found across multiple studies, by the same group based in Argentina, to exert beneficial effects. It was studied in depth using both in vivo and in vitro models. *Lb. plantarum* CIDCA 83114 was observed to have a protective effect against pathogen invasion of cultured human cells, with pre-incubation of the strain with Caco-2 or HT-29 colon cell lines resulting in a significant reduction in the internalization of the pathogen *Shigella flexneri* [6]. Of particular note is the fact of its protective effects in *both* cell lines, a property not observed in other strains tested in this study. HT-29 cells were also transfected with a plasmid expressing GFP as a reporter of NF-κB induced inflammation, as previous studies have shown that *S. flexneri* induces several markers of acute inflammation via the NF-κB pathway [79]. Notably, pre-incubation of HT-29 cells with the CIDCA 83114 strain approximately halved the *S. flexneri*-induced NF-κB activation of these cells.

This group also completed an in vivo study using hamster models (*Mesocricetus auratus*) [5], due to its relevance as a model of human infection by *Clostridium difficile* [80,81]. *Lb. plantarum* CIDCA 83114 was used as part of a microbial mixture (MM) of several bacterial and yeast species administered to hamsters in drinking water (using different dilutions at rates of 1:100 and 1:1000) prior to infection with *C. difficile*. It was found that treatment with the 1:1000 MM significantly reduced the proportion of animals with diarrhea and mortality. Interestingly, the 1:100 MM dilution result in no improvement in symptoms, suggesting too high a dosage of microbes may be detrimental, possibly exacerbating any inflammatory response to *C. difficile*. It was found that in mice treated with clindamycin alone, a significant number of animals suffered from diarrhea, with a number also dying. Administration of 1:1000 MM reduced the damage caused by clindamycin administration, suggesting kefir consumption could be used as a co-treatment along with antibiotic treatment, in addition to a prophylactic role. Histological analysis of cecal tissue also revealed that in animals treated with *C. difficile* and clindamycin, thickening of the mucosa, edema, inflammatory infiltrate with a predominance of neutrophils, and cryptic abscesses were observed. In animals treated with clindamycin alone, chronic inflammation with an infiltration of lymphocytes was observed. In animals treated with 1:1000 MM followed by clindamycin and *C. difficile* infection, no acute colitis occurred. Although mild thickening of the mucosa, along with slight infiltration of lymphocytes, was noted, in comparison to the previous two groups, the histological damage was significantly reduced. This study also included an in vitro analysis to test if the protective effects were in any way related to the effects of TcdA and TcdB (*C. difficile* toxins). The marker for toxin activity used was a cell detachment ratio. The assay found that fecal filtrate extracted from the animals treated with the 1:1000 MM had low levels of cell detachment, comparable to levels seen in control animal faecal filtrate, which had no exposure to *C. difficile* toxins. These levels were significantly lower than those observed following exposure to fecal filtrate extract from animals treated only with *C. difficile*.

Another group tested the effect of various potential probiotic strains, including *Lb. plantarum* CIDCA 83114, on *S. flexneri* invasion in Hep-2 cells [7]. It should be noted that Hep-2 cells originate from a laryngeal carcinoma, and not the intestine. This experiment tested the relative effect of pre-incubation or co-incubation of Hep-2 cells with various microbial strains found in kefir against *S. flexneri*, and found that, in general, pre-incubation of Hep-2 cells exerted a significantly higher protective effect than co-incubation. Nevertheless, a co-incubation assay conducted with *Lb. plantarum* against *S. flexneri* resulted in a significant reduction in pathogen invasion (5% vs. 100% in the control). In contrast, other strains brought about only very modest reductions compared to the 100% control (*Lb. kefiri* CIDCA 8348: 99%, *Lc. lactis* CIDCA 8221: 97%, *Sac. cerevisiae* CIDCA 8112: 89.3%, *K. marxianus* CIDCA 8154: 88.6%). Pre-incubation of the Hep-2 cells resulted in an improved performance from all potentially-probiotic strains, with the level of internalization with *Lb. plantarum* CIDCA 83114 being improved to 3.7% vs. the 100% control. Although the effects were less successful, other strains also performed well when assessed using the pre-incubation assay (*Lb. kefiri* CIDCA 8348: 68.4%, *Lc. lactis* CIDCA 8221: 42.6%, *Sac. cerevisiae* CIDCA 8112: 45.3%, *K. marxianus* CIDCA 8154: 32.2%). The pre-incubation dose of *Lb. plantarum* CIDCA 83114 was also tested for its effect on *S. flexneri* and *S. sonnei* invasion. For *S. flexneri*, 10^9^ CFU/mL CIDCA 83114 resulted in 3.7% internalization, as stated above. A lower dose of 10^8^ CFU/mL resulted in the treatment being less effective, leading to 12.7% internalization. Thus, for modulating cell invasion by *S. flexneri*, the treatment is dose dependent at these levels of dosage [7]. However, when the assays were performed with the same levels tested for *S. sonnei*, there was very little difference in the level of internalization, with treatment at 10^8^ CFU/mL resulting in an 8.7% internalization, and 10^9^ CFU/mL resulting in an 8.4% internalization, i.e., a very modest, non-significant improvement. Thus, prevention of *S. sonnei* cell invasion was not dose dependent at this level. However, it significantly reduced the internalization of *S. sonnei* and demonstrated the ability of *Lb. plantarum* CIDCA 83114 to reduce internalization of more than one pathogen.

*Lb. plantarum* CIDCA 83114 was also assayed as part of a two-strain and five-strain mixture [7]. Internalization of both *Shigella* strains were significantly reduced compared to the individual strains tested, suggesting the strains worked synergistically to exert improved positive effects. Scanning electron microscopy of the morphological changes seen on the surface of Hep-2 cells in response to various treatments was also performed. Treatment with *S. flexneri* resulted in removal of microvilli, cellular retraction, and protrusion formation along the cell surfaces. However, pre-treatment of Hep-2 cells with the five-strain mixture prevented any morphological abnormalities induced by *S. flexneri* infection. Furthermore, treatment of Hep-2 cells with the mixture alone did not lead to any morphological changes, indicating its safety [7]. The authors wished to analyze the mechanisms of the effects observed, and so isolated the cell wall fraction of *Lb. plantarum* CIDCA 83114 at concentrations equivalent to the levels present in the five-strain mixture (10^9^ CFU/mL), and preincubated the Hep-2 cells with this fraction [7]. This treatment significantly reduced the internalization (30% vs. 100% control) of both *Shigella* strains by approximately the same amount. However, the effect was diminished relative to the level of reduction achieved with intact *Lb. plantarum* CIDCA 83114 cells (3.7% vs. 100% control in *S. flexneri*, 8.4% vs. 100% control in *S. sonnei*). Nonetheless, a notable proportion of the reduction can thus be attributed to the cell wall fraction, and, thus, it is clear the cell wall plays a crucial role in exerting such effects. It was also noted that the dose of the cell wall was also significant. Cell wall extracted from a concentration of 10^12^ CFU/mL cells led to roughly 15% internalization of both *Shigella* strains vs. 100% control, and thus, the treatment was dose dependent at these levels [7]. Treatment of intact *Lb. plantarum* CIDCA 83114 cells with pepsin significantly reduced the protective effect (roughly 90% internalization vs. 100% control in *S. flexneri*, roughly 75% internalization vs. 100% control in *S. sonnei*), adding weight to the suggestion that key cell wall proteins/peptides play a crucial role in exerted the effects observed. 

In a separate study, this group also examined the ability of *Lb. plantarum* CIDCA 83114 to protect Vero cells from the effects of *Escherichia coli* O157:H7 strain 69160 supernatant containing type-II Shiga toxin [3]. It should be noted that Vero cells are not of intestinal origin, originating from a kidney epithelial cell line. Incubation of Vero cells with *E. coli* 69160 supernatant resulted in a significant reduction in cell viability. However, coincubation of these cells with *Lb. plantarum* CIDCA 83114 led to a significant reduction in loss of cell viability [3]. This group also tested the mechanism by which these effects were exerted [3] by testing the effect of intact cells vs. the cell wall fraction of this strain in a cell viability assay. It was found that both equivalently increased the viability of cells co-incubated with *E. coli* supernatant [3]. Thus, it is likely that the cell wall of CIDCA 83114 plays a crucial role in conferring the anti-cytolytic benefits seen in this strain. Further testing found that the reduction in cytotoxicity conferred by CIDCA 83114 was completely abolished by heating the CIDCA 83114 cells to 100 °C for 10 min, suggesting the key proteins in this process are heat sensitive [3]. Furthermore, coincubation with the proteolytic enzymes pepsin, proteinase K, and chymotrypsin resulted in a reduction in the protective effect observed in previous assays. Interestingly, coincubation with trypsin did not result in any reduction, thus indicating that the key proteins are resistant to this enzyme. Additional testing revealed that *E. coli* supernatant co-incubated with CIDCA 83114 had a reduced level of the *E. coli* toxin Stx, as seen by the absence of a band corresponding to the B subunit of that protein on a Western Blot [3]. The resulting supernatant was then tested against Vero cells and was found to have a reduced cytotoxic effect compared to untreated *E. coli* supernatant. This suggests that binding or proteolysis of Stx by proteins found in the cell wall of CIDCA 83114 is at least partly responsible for the protective benefits observed.

Another study investigated the protective effect of *Lb. plantarum* CIDCA 83114 against pathogenic *E. coli* [9]. In the cell adhesion assay, it was found that pre-treatment with 10^8^ CFU L/plantarum cells/well was only effective in lowering adhesion of *E. coli* cells if the concentration of the pathogen was less than 10^6^/well. Treatment of the Hep-2 cells with CIDCA 83114 at higher concentration of pathogen (10^7^ and 10^8^) offered no significant difference vs. no treatment. Thus, according to the authors, the ratio of *Lactobacillus* present needs to be at least 1:100 in order to exert protective effects. In the cell detachment assay, it was found that over 60% of Hep-2 cells detached when incubated with *E. coli*. However, when the Hep-2 cells were pre-incubated with CIDCA 83114, the cell detachment was significantly reduced to 20%. Furthermore, UV-killed CIDCA 83114 had very similar protection values to its viable counterparts, indicating it is not crucial that the *Lactobacillus* be viable. The morphological structure of the Hep-2 cells was also analyzed, using immunofluorescent tagging and scanning electron microscopy. Disorganization of the actin network, cell rounding, and cell retraction was observed in Hep-2 cells treated with *E. coli*. However, the extent of these alterations was almost completely abolished in the cells pre-treated with *Lactobacillus*.

#### 2.1.2. *Lactobacillus Plantarum* C4

*Lb. plantarum* C4 has also been studied in depth. This strain exhibited significant adhesion to Caco-2 cells, as well as antimicrobial activity against *L. monocytogenes*, *Y. enterocolitica*, *S. typhimurium*, and *E. coli* in a spot assay test [23]. The authors also conducted in vivo experiments in mice. After administration of this strain (at 9.4 × 10^9^ CFU/mouse), levels of C4 were detected in the feces after one day, but detectable levels quickly decreased following this. The immunomodulatory effects were also analyzed. For this, the mice were administered cyclophosphamide (CP) in order to stimulate myelotoxic effects [23]. Treatment with C4 did not reverse the lower levels of peripheral blood leukocytes or spleen cells induced by CP treatment, but the proliferation of splenocytes, which was nearly abolished by CP treatment, was partially restored by C4 treatment [23]. 

In a further paper by this group [24], it was shown that CP treatment resulted in a proliferation of segmented filamentous bacteria (SFB) in the gut of the mice, a feature that does not normally occur in adulthood. However, mice treated with 1 × 10^10^ CFU of *Lb. plantarum* C4 had levels of SFB comparable to healthy controls. Even in healthy, non-immunocompromised mice, treatment with C4 also significantly reduced levels of SFB, suggesting that this strain has a potential effect in modulating host response to certain bacteria or altering the composition of the microbiome themselves. 

This group conducted another study, investigating the effect of *Lb. plantarum* C4 treatment in mice later infected with *L. monocytogenes* [26]. Mice were administered 5 × 10^7^ CFU of the strain via gastric intubation daily for four weeks. They were then challenged with *L. monocytogenes* infection. Pre-treatment with C4 led to increased survival of the infected mice compared to non-treated controls. However, it was not successful in clearing the pathogen from the spleen or liver, with levels of the pathogen in these organs being similar to non-treated controls. Pre-treatment with C4 also had effects on a number of pro-inflammatory cytokines following *L. monocytogenes* infection. In the treated mice, compared to untreated controls, IL-1 and IL-6 were present in significantly higher levels in serum on day 2, before and after which, levels flatlined. However, in untreated mice, IL-1 levels underwent a gradual increase, peaking at day 4 before returning to base levels. With regard to IL-6, levels gradually decreased following a peak on day 2. Together, this may suggest that *Lb. plantarum* C4 primes the immune system to bacterial infections. Pre-treatment had no effect on the levels of TNF-α as compared to untreated controls.

This strain was further tested in a later study by these authors [27]. They confirmed the previous observation that the strain inhibited the growth of both *L. monocytogenes* and *E. coli*, as seen via a spot assay test. Higher concentrations of the spent culture supernatant (SCS) of C4 was also effective in inhibiting growth of *E. coli*. The SCS of C4 was effective in permeabilizing the cell envelope of *E. coli*, as evidenced by high fluorescence when treated with sytox green, a high-affinity nucleic acid stain. Interestingly, the treatment with SCS of this strain also led to very significant levels of cell death amongst HL-60 cells, a human leukemia cell line. This supernatant also exhibited high cytotoxicity (demonstrated by lactate dehydrogenase enzyme release) against these cells, leading the authors to suggest potential anti-carcinogenic effects. However, the SCS of this strain also exhibited high hemolytic activity, which is more consistent with non-specific cytotoxicity. 

In further experimentation, pre-treatment of mice with this strain was shown to promote clearance of *Y. enterocolitica* from Peyer’s patches [22]. Pre-treatment with C4 also appeared to prime immune cells to release TNF-α in response to *Y. enterocolitica* infection, with fecal levels being higher in these mice compared to untreated controls. However, such a phenomenon was not replicated in the plasma of these animals, suggesting a local immune response. Infection with this pathogen also significantly reduced fecal levels of IL-10 in untreated control animals. However, levels of IL-10 were not different between infected and un-infected animals when these animals had been treated with C4. Interestingly, among un-infected animals, treatment with C4 led to a significant decrease in IL-10 levels compared to un-treated controls. There was also a much higher level of secretion of IgA in animals treated with C4 in response to pathogen infection, as compared to untreated controls, suggesting that this strain may have a role in priming the immune system to pathogens. 

#### 2.1.3. *Lactobacillus Plantarum* MA2

The strain *Lb. plantarum* MA2, isolated from Tibetan kefir grains, was studied in depth in a series of papers from the same lab. This included an in vivo study in rats [20]. These rats were fed lyophilized *Lb. plantarum* MA2 powder at a daily dose of 10^11^ cells/day/rat. At the end of a five-week trial, total cholesterol (TC), low-density lipoprotein cholesterol (LDL-C), and triglycerides (TG) were all significantly lower in the treated rats, with high-density lipoprotein cholesterol (HDL-C) showing no difference. These rats also exhibited significantly lower liver TC and TG levels, as well as significantly higher fecal weight and fecal cholesterol levels. The treated mice also showed significantly increased levels of lactic acid bacteria, as expected from the very high dosage received. Interestingly, however, levels of bifidobacteria were also significantly increased. Furthermore, both bifidobacteria and lactobacilli increased in relative abundance in comparison to control animals after a relatively similar amount of time, i.e., between days 20–24. This would seem to indicate that introducing lactobacilli may alter the intestinal environment in a manner that favors growth of bifidobacteria.

The same group later investigated the antioxidative effects and colonization ability of this strain in vivo [19]. Mice were treated with D-galactose in order to induce an aging model. Randomly selected mice were then treated with various levels of the strain, high at 10^10^ CFU/day, medium at 10^9^ CFU/day, and low at 10^8^ CFU/day. Bloods were then taken after six weeks. It was found that treated mice had lower levels of malondialdehyde (MDA), a marker for oxidative stress, and this occurred in a dose-dependent manner. MDA levels were also significantly reduced in the liver. However, treatment with this strain resulted in no change in the total antioxidant capacity (T-AOC). Glutathione peroxidase (GSH-Px) activity was upregulated in the blood and liver of treated mice, as was superoxide dismutase (SOD), with this activity being dose dependent in the liver. MA2 antioxidant effects were also demonstrated in vitro by the same authors [18]. With regard to colonization, the authors used a bioluminescent tag attached to the bacterial cells administered. The strains’ bioluminescence levels dropped off significantly once treatment was stopped, either after a single dose of 3 × 10^8^ CFU or a continuous two-week daily dose. However, microbiological analysis of feces retrieved from the colon and ileum was performed via serial dilution and revealed that MA2 was still present in sufficient quantities (10^4^–10^5^ CFU/g) to maintain colonization 21 days after oral administration of the strain. Furthermore, this strain showed high-level auto-aggregation, considered an important characteristic in potential probiotics [17]. 

#### 2.1.4. Other *Lactobacillus Plantarum* STRAINS

As per the 2013 Kakisu study above [3], it was also found that coincubation of Vero cells with *Lb. plantarum* CIDCA 8336 led to a significant reduction in the loss of cell viability of Vero cells exposed to *E. coli* 69160 supernatant.

Another group investigated in depth the characteristics of *Lb. plantarum* B23 [10]. This strain exhibited high-level adhesion to Caco-2 cells. It was susceptible to a range of antibiotics. However, it was found to be resistant to tetracycline. The authors later performed in vivo studies, analyzing the serum lipid levels of rats treated with daily doses of 10^9^ CFU/mL of the selected strain. They reported significantly lower levels of TC, TG, LDL-C, and significantly higher levels of HDL-C. Rats fed this strain also had lower levels of liver cholesterol content, and correspondingly higher levels of fecal cholesterol content [10]. Additionally, this strain colonized the gut at various points, particularly in the ileum, colon, and feces. Interestingly, after two weeks of stopping the treatment, the strain was still found in the small intestine, colon, and feces, with levels only having slightly declined [10].

A number of different strains that also show potential health benefits have been studied by the Huang group. These include *Lb. plantarum* Lp27 [11], Lp09, and Lp45 [14]. Lp27 has high-level adhesion to Caco-2 cells, a desirable characteristic in potential probiotics. It is also susceptible to a range of antibiotics, meaning it could easily be eradicated if required, an additional safety reassurance. Animals administered all strains underwent blood lipid analysis, whereby blood samples were taken from rats treated with a daily dose of 10^9^ CFU/mL of their respective strain over the course of four weeks. All strains reduced the levels of TC, TG, and LDL-C. However, HDL-C levels were unaffected. Of these strains, Lp27 and Lp09 had the greatest effect and these strains also reduced the levels of TC and TG in the liver. Furthermore, Lp09- and Lp45-treated animals had higher fecal cholic acid levels compared to untreated controls. Investigating the mechanism by which such effects may occur, the authors found that Caco-2 cells incubated with Lp27 had a micellar cholesterol uptake approximately 1.5-fold lower than of untreated control cells. Furthermore, they found that Lp27 significantly downregulated the expression of Niemann-Pick C1-like 1 (NPC1L1; a protein that is critical for intestinal cholesterol absorption) mRNA in Caco-2 cells as well as in ex vivo explants of the small intestine.

A paper published by a different group highlighted the anti-oxidant effect of an exopolysaccharide (EPS) produced by *Lb. plantarum* YW11 [13]. The EPS isolated from this strain showed high scavenging activity against hydroxyl radicals and superoxide anions. In in vivo studies conducted in mice, high doses of this EPS relieved oxidative stress in aging mice (induced by D-galactose) accompanied by increased levels of glutathione peroxidase, superoxide dismutase, catalase, and higher overall antioxidant capacity. Treatment with EPS recovered some of the microbial gut diversity, which was reduced by D-galactose treatment, decreasing levels of pathogenic *Flexispira* (37.5 fold), whilst increasing levels of *Blautia* (36.5 fold) and *Butyricicoccus* (9.5 fold), which correspondingly decreased nitrogen oxide levels and increased short-chain fatty acid levels.

A number of studies have identified strains with antimicrobial activity. The SCS of *Lb. plantarum* ATCC 10012 was found to inhibit growth of both *Streptococcus mutans* and *Streptococcus sobrinus*, as well as inhibit formation of biofilm [15].

*Lb. plantarum* CIDCA 8327 was shown in one study [8] to have an inhibitory effect on the growth of *E. coli*, *Sal. enterica* serovar Enteritidis, *Sal. enterica* serovar Tiphymurium, *Sal. gallinarum*, and *Sh. sonnei*, the only strain of a range of lactobacilli to have an inhibitory effect on all pathogens tested. This inhibition was tested by using a spot test assay. 

*Lb. plantarum* CIDCA 8337 displayed very high adhesion to Caco-2 cells. In a spot agar assay, it also demonstrated significant inhibition of *E. coli* and *S. typhimurium* [8].

#### 2.1.5. Studies of Unspecified *Lb. plantarum* Strains

Finally, an unspecified *Lb. plantarum* strain, as part of a mixture contain six lactic acid bacterial strains, was found to enhance the cytotoxicity of NK cells against human chronic myelogenous leukemia K562 cells and colorectal tumor HCT116 cells [29]. Additionally, secretion of IFN-γ was increased in these NK cells, along with increased mRNA expression. 

### 2.2. Lactobacillus kefiranofaciens

#### 2.2.1. *Lactobacillus Kefiranofaciens* M1

Across multiple studies, the strain *Lb. kefiranofaciens* M1 has been found to exert some antiallergenic-related effects regarding Th1/Th2 balance. In an early study [37], *Lb. kefiranofaciens* M1 cells were found to significantly stimulate IL-6 production in a murine macrophage cell line (RAW 264.7). Interestingly, when just the supernatant from this species was tested, it was found to significantly stimulate IL-6, TNF-α, IL-1β, and IL-12 at higher levels than obtained by the cells alone. However, in a later assay undertaken in a different murine peritoneal macrophage cell line, the cell responses were reversed, with treatment with the bacterial cells resulting in higher concentrations of cytokines compared to the supernatant. This highlights the challenge of relying on cell-line based analysis of health-promoting properties in gut commensals and the resulting interpretations that persist in the literature in this field. Regardless, *Lb. kefiranofaciens* M1 certainly had an effect on production of a number of cytokines. In the same paper, the authors partially identified the mechanism of IL-6 stimulation by *Lb. kefiranofaciens* M1 cells and their supernatant by knocking out the TLR-2 receptor. This resulted in a significant decrease in IL-6 production from *Lb. kefiranofaciens* M1 cells. However, IL-6 production resulting from its supernatant was unaffected, suggesting that the mechanism of action in this instance involved a separate receptor.

A later study from the same group further demonstrated the cytokine inducing ability of *Lb. kefiranofaciens* M1, which induced the production of IFN-γ, TNF-α, IL-6, IL-12, and IL-1β in murine peritoneal macrophage cells [33]. Other than TNF-α and IL-1β, there were no differences in the responses elicited by live and heat-inactivated cells. Live strains stimulated higher cytokine production of IL-1β, while heat-inactivated strains caused higher-level production of TNF-α. This study also demonstrated that heat-inactivated *Lb. kefiranofaciens* M1 significantly reduced the levels of serum IgE in vivo, and that this effect was dose dependent. This effect was also demonstrated over a time period of four weeks. In an effort to uncover the mechanism behind such effects, the authors investigated the levels of certain cytokines expressed in splenocytes from the mice studied. There was a significant reduction in the levels of IL-5, with a corresponding increase in the levels of IL-12. This led the authors to suggest that *Lb. kefiranofaciens* M1 regulates the Th1/Th2 balance, upregulating the Th1 pathways while downregulating the latter [33]. It was later found that the treatment with *Lb. kefiranofaciens* M1 upregulated CD4^+^CD25^+^ regulatory T-cells, which downregulated Th2 cytokines [33]. Levels of CD19 were reduced and, because this is a marker for B cell lineage, suggests reduced B cell proliferation, which likely had an influence on the decreased levels of IgE expression. The authors also investigated the effect of *Lb. kefiranofaciens* M1 at the host response level, measuring expression of various genes in the Peyers’ patches of the treated mice. They found an upregulation of genes involved in immune responses, inflammation, and cell adhesion, along with a decrease in expression of genes associated with the classic complement pathway and lectin-induced pathway [33].

A further study by this group investigated the effect of *Lb. kefiranofaciens* M1 on the allergic airway response in mice [34]. A number of aspects were tested, such as the duration and dosage of the treatment provided. One group of mice that were administered a 10^8^ CFU/mL dose every day for 32 days (Course A) displayed significant reductions in the levels of IL-4, IL-13, IL-6, IL-1β, CCL20, and TNF-α. Mice who had been administered with the same dose for the first 14 days of the 32 days course (Course B) and the last 3 days of the 32 day course (Course C) displayed significantly decreased levels of IL-6 and TNF-α. Course A also displayed a significant increase in the levels of T-reg cell numbers. However, as demonstrated by course B, these benefits are not permanent, and, thus, it is apparent that efforts need to be made to continuously administer microbial treatments. Additionally, course C demonstrated that short-term consumption is not effective. On this basis, the authors continued their experiments exclusively with the Course A treated animals [34]. They demonstrated that the effects observed were dose dependent, with a trend towards graduated decreases in the levels of various cytokines (IL-4, TNF-α, IL-5, IL-6, IL-1β, IL-17). *Lb. kefiranofaciens* M1 was also able to significantly reduce levels of IgE. Histological changes were also noted. Treatment with 10^8^ and 10^9^ CFU/mL resulted in significantly lower levels of eosinophil infiltration in the lung parenchyma. Such treatment also abolished the overproduction of mucus and goblet cell hyperplasia detected in asthma-induced mice. Furthermore, the beneficial effects were greatest in the mice treated with the higher dose [34].

This group also investigated the effects of *Lb. kefiranofaciens* M1 in in vitro and in in vivo models of experimental colitis [32]. Initially, they analyzed the effects of *Lb. kefiranofaciens* M1 on the transepithelial electrical resistance (TEER) on an epithelial monolayer of Caco-2 cells, as a measure of epithelial integrity. Treatment with *Lb. kefiranofaciens* M1 resulted in an increase in epithelial barrier integrity, which was dose dependent. However, only the highest dose (10^8^ CFU/mL) showed a significant change in epithelial barrier integrity vs. the control of no dose. Treatment of Caco-2 cells with *Lb. kefiranofaciens* (10^8^ CFU/mL) also upregulated the chemokine CCL-20, which mediated the restitution of colonic epithelial cells. However, other similarly acting chemokines (CXCL-12 and IL-8) did not undergo a significant change. Treatment of the monolayer with *Lb. kefiranofaciens* cells also resulted in accelerated recovery of membrane integrity following dextran sulfate sodium treatment. This encouraged the researchers to test this model in vivo. Mice fed the *Lb. kefiranofaciens* at 10^8^ CFU/mL had lower rectal bleeding scores and increased colon length compared to controls during DSS induced colitis. Histology also indicated a loss of goblet cells and disturbed mucosal architecture in controls, which was significantly ameliorated in treated mice. The treated mice had a significantly lower histological score (indicating healthier histological features) compared to control mice. Ex vivo colon samples were also assessed for cytokine production. Levels of IL-1β and TNF-α, pro-inflammatory cytokines, were both significantly reduced in the treated mice, while IL-10, an anti-inflammatory cytokine, was found to be significantly higher in treated mice. This group also investigated the role of TLR2 in the protective effects of *Lb. kefiranofaciens* treatment. The amelioration of rectal bleeding and colon length observed in mice treated with *Lb. kefiranofaciens* was not observed in the TLR2 knockout mice. Furthermore, *Lb. kefiranofaciens* treatment lead to a significant upregulation of NFκB when TLR2 was present in TLR2-lacking HEK-293T cells. When TLR2 was blocked, the increase of CCL20 induced by *Lb. kefiranofaciens* treatment was significantly decreased.

In a later study, the same authors went on to conduct further experimentation in in vivo models [30]. Continuous intragastric administration of *Lb. kefiranofaciens* M1 resulted in increased ileac villus length/crypt depth and increased levels of goblet cells as compared to germ free (GF) and single oral inoculation treated mice. Treated mice also had improved intestinal bleeding scores in response to DSS-induced colitis when compared to non-treated mice. Histologically, there was also a significant difference between mice treated with *Lb. kefiranofaciens* vs. untreated mice when tested against DSS-induced colitis, with a lowering in the histological score from 9 (untreated mice) to 2 (treated mice). This paper also showed that *Lb. kefiranofaciens* enhanced the immune response to TLR agonists, such as lipopolysaccharide (LPS) and R848, with IFN-γ and IL-12 levels significantly higher in treated mice relative to GF controls [30]. It should be noted, however, that this is not surprising in that any bacterium could be expected to elicit a stronger response than GF controls. It was also noted that it was unlikely that *Lb. kefiranofaciens* M1 colonized the gut after single oral inoculation, given that fecal levels dropped significantly by day 5 and being undetectable past day 7 [30]. Additionally, *Lb. kefiranofaciens* was unable to adhere to Caco-2 cells in vitro. Together, along with the previous data, this suggests that this strain is unable to colonize the gut and needs to be taken continuously to see the observed effects. However, it is likely that total confirmation could only result from an intestinal swab, not feces analysis alone.

The same authors researched the effect of *Lb. kefiranofaciens* M1 treatment on enterohemorrhagic *Escherichia coli* (EHEC) infection [31]. Treated mice were administered 2 × 10^8^ CFU/mL of *Lb. kefiranofaciens* intragastrically for seven days, while controls were administered phosphate buffered saline (PBS). The mice were then challenged with EHEC infection at various timepoints. Pre-treatment with the *Lb. kefiranofaciens* strain prevented a decrease in food intake, which was caused by EHEC infection. Additionally, intestinal bleeding was significantly reduced in the Lb-treated group compared to the controls. The histological scores were also improved in the Lb-treated mice, with reduced atrophy of the villi, as well as an amelioration of the loss of crypts and Paneth cells. Tissue edema and loss of epithelial integrity were reduced following *Lactobacillus* pre-treatment. Interestingly, pre-treatment with *Lb. kefiranofaciens* also resulted in a near absence of congestion of the renal glomeruli and kidney interstitial tissue, which was severe in the untreated but EHEC-infected mice. Heat treatment of the *Lactobacillus* strain reduced its effectiveness. Pre-treatment of the mice with the *Lactobacillus* strain also reduced translocation of *E. coli* to the liver and spleen, and *E. coli* could not be detected in the blood of pre-treated mice, whereas significant numbers were found in the un-treated controls. Correspondingly, serum levels of EHEC virulence factors Stx-1 and Stx-2 were significantly reduced in in the *Lb. kefiranofaciens* M1 pre-treated mice. Pre-treatment also resulted in increased levels of EHEC specific IgA in the feces compared to untreated controls. However, these increases were only detected in the feces, and were not systemic, suggesting a local immune response. Using in vitro intestinal cell models, the researchers determined that pre-treatment of Caco-2 cells with the *Lactobacillus* strain decreased levels of cell death when subsequently challenged with EHEC infection. Furthermore, this protection operated in a dose-dependent manner, and was reduced when the *Lactobacillus* strain was heat-treated. Pre-treatment also improved intestinal cell monolayer integrity as measured by TEER. However, pre-treatment was essential. Similar effects were not observed when the strain was co-cultured with the EHEC cell-free supernatant, nor did the co-culture method reduce levels of Stx-1/Stx-2 in the EHEC cell-free supernatant.

In a later study, the oral toxicity of *Lb. kefiranofaciens* in rats was evaluated [35]. A high dose of 1.8 × 10^10^ CFU/kg was used. At this high dose, when compared to controls, no abnormalities or adverse effects were observed. A number of parameters were assayed, including body weight, common serum biochemistry and hematology markers, as well as terminal organ weights. Organs and tissues were also histologically examined. Together, no adverse effects were observed with this high dose, indicating the regular dose of around 2 × 10^8^ CFU/mL would appear to be within a safe range. However, it should be noted that such experimental observations have not been tested in human subjects. That being said, *strain-specific* consumption of kefir-isolated bacteria is unheard of and unlikely to occur. Nonetheless, such toxicity testing may arguably be warranted, especially if kefir lactobacilli were developed as single-strain probiotics. 

#### 2.2.2. Other *Lactobacillus kefiranofaciens* Strains

An early study identified *Lb. kefiranofaciens* 10058 as displaying a number of potentially beneficial properties [46]. This strain inhibited growth of all the pathogenic bacteria tested (*E. coli*, *L. monocytogenes*, *S. typhimurium*, *S. enteritidis*, *S. flexneri*, *Y. enterocolitica*), as determined using an agar spot test. It also showed high-level adhesion to Caco-2 cells. This strain was also shown to produce a kefiran-related exopolysaccharide. Kefiran has been shown in previous studies to retard tumor growth [82].

Another strain found to have potential health benefits is *Lb. kefiranofaciens* DD2 [15]. SCS of this strain significantly inhibited the growth of two oral pathogens (*S. mutans* and *S. sobrinus*) in nutrient broth. It also significantly inhibited the biofilm formation by the pathogens. Furthermore, it influenced the expression of several genes in *S. mutans*, with mRNA expression levels of proteins encoding for carbohydrate metabolism, adhesion, and other regulatory mechanisms being significantly reduced by this strain, when *S. mutans* cells were co-cultured with the SCS of *Lb. kefiranofaciens* DD2.

A further study by the same group on a related strain also reported beneficial properties [48]. *Lb. kefiranofaciens* DN1 was found to increase the weight of feces and its water content in mice that were treated with a daily 2 × 10^8^ dose for two weeks. Together, this is suggestive of a possible role in treating constipation or improving intestinal mobility. Furthermore, it altered the composition of the microbiome of the mice treated, with *Firmicutes*, *Bacteroidetes*, *Lactobacillus*, and *Prevotella* being found at significantly higher levels. Furthermore, opportunistic pathogens such as *Proteobacteria*, *Enterobacteriaceae*, and *Clostridium* were less abundant. It was also shown to produce very high levels of a novel EPS [83] This purified EPS exhibited the ability to significantly reduce the levels of *L. monocytogenes* and Salmonella Enteritidis, and this ability was dose dependent.

*Lb. kefiranofaciens* KCTC 5075 displayed anti-inflammatory characteristics (as part of a three strain mix also containing *Lb. kefirgranum* KCTC 5086 and *Lb. kefiri* KCTC 3611 EVs) whereby its extracellular vesicles (EV) reduced levels of IL-8 in Caco-2 cells exposed to TNF-α [49]. Treatment with this strain also significantly improved the body weight, rectal bleeding, and stool consistency of mice with 2,4,6-trinitrobenzenesulfonic acid-induced inflammatory bowel disease. Treated mice also had an improved histopathological score and reduced myeloperoxidase activity, showing they were less inflamed. 

In an earlier study, an exopolysaccharide produced by *Lb. kefiranofaciens* ATCC 43761 was also analyzed for its potential health benefits [51]. This in vivo trial was conducted in mice who had been administered exopolysaccharide at a dosage of 100 mg/kg body weight/day for two, five, or seven consecutive days. Treatment with the exopolysaccharide at any level led to significant increases in the levels of IgA positive cells in the small intestine. Furthermore, after five days of treatment, the levels of IL-6 and IL-12 positive cells were significantly increased. This increase was also seen for IL-10 positive cells, but only after seven days. However, regarding the large intestine, these changes were detected in an even quicker time-frame of only two days (except for IL-12, which was not assayed in the large intestine). Interestingly, these changes were also observed for TNF-α, and to a lesser extent IFN-γ. Serum levels of IL-4, IL-10, and IFN-γ were increased after seven days, whereas IL-6 was significantly increased after two days.

#### 2.2.3. Studies of Unspecified *Lb. kefiranofaciens* Strains

A number of papers have reported on other benefits relating to this species, while not specifying a particular strain or strains.

For example, one group described the effects of kefiran, a product of *Lb. kefiranofaciens* [44]. Rabbits were fed a high-cholesterol diet with or without kefiran every day for eight weeks. The total area of atherosclerotic lesions was reduced compared to controls, with the biggest improvements being seen at points in the aorta furthest from the heart. The liver also exhibited significantly lower levels of TC. 

Kefiran was also investigated for its immunomodulatory effects in bone marrow-derived mast cells (BMMCs) [43]. Pre-treatment with kefiran reduce levels of degranulation by mast cells. It also significantly reduced TNF-α secretion.

Another study investigated the effect of *Lactobacillus*-derived extracellular vesicles (EVs) in a mice model of inflammatory bowel disease (IBD) [49]. The EVs were derived from different Lactobacillus species, including *Lb. kefiranofaciens*, *Lb. kefiri*, and *Lb. kefirgranum*. Confirmation that these vesicles were taken up by Caco-2 cells was provided by immunofluorescent microscopy. The *Lb. kefiranofaciens* EVs significantly reduced the mRNA expression levels of IL-8 in TNF-α stimulated Caco-2 cells. In the in vivo section of this study, mice were administered these EVs daily for 10 days, at either 3 × 10^8^ CFU/mL. Half the mice were also induced with a model of inflammatory bowel disease by 2,4,6-trinitrobenzene sulfonic acid treatment. Mice treated with EVs showed a reduction in loss of body weight when compared to controls. They also suffered less rectal bleeding and had improved stool scores. Furthermore, these mice also had significantly healthier histological scores [49]. 

### 2.3. Lactobacillus kefiri

#### 2.3.1. *Lactobacillus kefiri* CIDCA 8348

It has been shown that *Lb. kefiri* CIDCA 8348 had a high auto-aggregating capability, as well as strong coaggregation with *Salmonella* [57]. This strain also showed high-level adhesion to Caco-2 cells. Such characteristics are desirable in a potential probiotic [57]. It was also reported that the SCS of this strain, along with strains CIDCA 8321, CIDCA 83113, and CIDCA 8344, significantly reduced the levels of adhesion and invasion of Caco-2 cells by *Salmonella*. 

A different study researched the role of this strain as part of a microbial mixture in relation to its effect on infection of *C. difficile* in hamsters [5]. It was found that pre-treatment with this microbial mixture dramatically improved the survival of those later infected with *C. difficile*. Furthermore, a wide range of histological changes induced by *C. difficile* infection were absent in the mice pre-treated with the microbial mixture. This group used the same microbial mixture to test for effects upon infection by *S. flexneri* [6]. They found that while the microbial mixture caused a significant reduction in the level of Shigella invasion into Caco-2 cells, the individual strain of 8348 did not. This observation was corroborated by another study [7]. However, only one strain of the microbial mixture had any significant effect individually. This could suggest that the strains act synergistically to bring about the greater significant effects seen with the microbial mixture. 

Further studies by this group showed that this strain caused a statistically significant effect on Vero cell viability when these cells were exposed to *E. coli* supernatant [3]. 

The same group found that surface proteins from *Lb. kefiri* CIDCA 8348 antagonized the cytotoxic effects of *Clostridium difficile* toxins in vitro [62]. This *Lactobacillus* strain was found to have an inhibitory effect on the ability of the SCS of *C. difficile* to induce cell detachment of Vero cells. This *Lactobacillus* strain also had a strong inhibitory effect on the *C. difficile* SCS’s ability to induce actin cytoskeleton disorganization. The researchers then determined the mode of action, by purifying the *C. difficile* toxins TcdA and TcdB. These toxins had a strong detachment effect in a cell detachment assay, which was significantly reduced by co-incubation with the surface proteins from the *Lactobacillus*. It was shown using immunofluorescent microscopy that these toxins are absorbed onto the surface of the *Lactobacillus*. Interestingly, neither heat treatment nor treatment with proteases reduced the ability of the surface layer proteins on the *Lactobacillus* to abrogate the negative effects of these *C. difficile* toxins. Only treatment with S-layer specific antibodies resulted in any reduction of the beneficial effects, suggesting that there is a direct interaction between the toxins and the surface layer proteins. Whether or not such a toxin-absorptive effect could occur sufficiently in a person infected with *C. difficile* remains to be seen.

A more recent study by the same group reported that this *Lb. kefiri* strain is able to adhere to mucus extracted from the small intestine and colon, highlighting this strain as an interesting candidate for probiotic selection [61]. They also found that this strain was able to inhibit growth of multiple different pathogens. They spotted a suspension of this *Lb. kefiri* strain onto an MRS agar plate, which was incubated for 24 h. Following this, various pathogens were seeded into soft BHI agar and plated over the spotted lactobacilli. Inhibition halos where then measured. Among the pathogens inhibited were *P. aeruginosa*, Salmonella enteritidis, *S. flexneri*, *B. cereus*, *L. monocytogenes*, *S. aureus*, and *E. faecalis*. However, such anti-microbial effects are common in lactic acid bacteria tested in this manner and may be due to production of lactate or hydrogen peroxide [61].

Safety testing of this strain established that it did not cause hemolysis and was susceptible to a wide range of antibiotics. Mice were also treated with a daily oral dose of 2 × 10^8^ CFU/mL, and no adverse health effects were observed, including no changes in levels of pro-inflammatory cytokines. Furthermore, no translocation of microorganisms to organs such as the liver or spleen was observed [59]. 

Based on these promising results, the researchers conducted an in vivo study, analyzing the effect of the strain on host immune response and gut microbiota [58]. Mice received a daily dose of 2 × 10^8^ CFU/mL of the strain for either 7 or 21 days. The controls received PBS. Continuous daily administration of the strain resulted in an increase in the levels of IgA in the feces after a period of 14 days. Expression levels of genes for relevant host response proteins were also examined following the administration of the strain. In the ileum, IL-1β and IL-17A expression was downregulated, while IL-10, CXCL-1, and mucin 6 were upregulated. In the colon, mucin 4 was upregulated while IFN-γ, GM-CSF, and IL-1β were downregulated. Gene expression was also examined in Peyer’s patches (PP) and mesenteric lymph nodes (mLN). In PPs, IL-23, IFN-γ, and IL-6 were downregulated. In mLN, IL-6, IL-23, IL-17A, GM-CSF, and RORγt transcription factor was downregulated, while IL-10 was upregulated [58]. Ex vivo experiments were conducted to analyze the response of ileum and colon tissue to LPS. LPS stimulated secretion of IL-6 and GM-CSF in these tissues. However, in treated mice, the increase in these secretions was significantly attenuated. Furthermore, treated tissue showed increased levels of secretion of IL-10 in response to LPS when compared to controls [58]. 

The same authors conducted research into the effect of this strain during consumption of a fructose-rich diet (FRD) [60]. Mice were administered a dose (10^8^ CFU/mL) every two days for six weeks. Mice on an FRD receiving this strain had significantly lower body mass compared to those not fed the *Lactobacillus*. This phenomenon was also observed with regard to triglyceride and leptin levels in plasma. Treatment with this strain also led to significantly lower levels of leptin mRNA expression in epidydimal adipose tissue compared to untreated controls. This pattern was also seen with mRNA expression of lipoprotein lipase (LPL) and hormone sensitive lipase (HSL). With regard to inflammation profile, this pattern was also seen with mRNA expression levels of IFNγ, IL-1β, and IL-6.

#### 2.3.2. Other *Lactobacillus kefiri* Strains

Pre-treatment of Vero cells with the S-layer proteins of *Lb. kefiri* CIDCA 83111 was reported to have a protective effect against SCS of *C. difficile* [62]. It was also shown to have an inhibitory effect on the growth of a wide range of pathogens (*P. aeruginosa*, Salmonella Enteritidis, *S. flexneri*, *L. monocytogenes*, *B. cereus*, and *S. aureus*) as assayed via a spot agar test [59].

*Lb. kefiri* CIDCA 83102 was shown to have a high-level aggregating ability, a desirable characteristic for a potential probiotic [52]. Co-incubating this strain with *S. enterica* significantly reduced the ability of the latter to infect Caco-2 cells. Pre-treatment of *Salmonella* cells with this *Lb. kefiri* strain also reduced the ability of the pathogen to infect cells, a trait not observed in many of the other strains tested, even amongst those that reduced infection when co-incubated with *S. enterica*. Pre-treatment with this strain also resulted in preservation of cytoskeletal architecture, which was seen to be disrupted when treated with *S. enterica*.

PFT is a natural mixture comprised mainly of freeze-dried *Lb kefiri* P-IF, also including trace amounts of three yeast strains. The authors of this study [68] analyzed this kefir product’s ability to activate dendritic cells (DC). What is particularly significant about this study is that it utilized donated human blood samples. PFT was found to upregulate the expression of DC surface co-stimulatory and maturation markers CD80, CD86, and HLADR, and these increases were dose dependent. It also induced production of cytokines IL-6, TNF-α, and IL-10 in DCs, with the latter being induced by a lower *Lactobacillus* cell number. Secretions of IFN-γ, TNF-α, and IL-10 were also increased from CD4^+^T cells. Blood DCs were also assayed, and it was found that their secretion of IL-29 and IFN-α were significantly increased. Blood DCs stimulated with PFT were incubated with CD8^+^T cells, which caused these T cells to upregulate CD107a and Granzyme-B expression. Both these markers are associated with cytotoxic T cells, which help the eradication of tumor cells and viral infected cells. PFT was further investigated by the authors in relation to its effect on cancer cells [67]. PFT was found to drastically increase levels of cell death amongst AGS cells (gastric cancer cell line). These cells also displayed morphological apoptotic characteristics. The specificity or biological relevance of this phenomenon is unclear.

*Lb. kefiri* DH5 was reported to have significant cholesterol reducing effects in vitro, and, thus, was selected for an in vivo study [70]. Mice were administered a dose of 2 × 10^8^ CFU of this strain daily for six weeks. At the end of the study, mice on a high-fat diet while being administered this strain (HFD-DH5 mice) had a significantly lower body weight compared to mice on a high-fat diet alone. These mice also had lower levels of TGs and LDL-cholesterol in their plasma. Interestingly, even those mice on a normal chow diet had lower levels of TGs if this strain was being administered. Macroscopic visual analysis of the livers of HFD-DH5 mice demonstrated similarity to those of mice on a regular chow diet. Histological analysis of hepatocytes of HFD-DH5 mice revealed similarities to those mice on a regular chow diet, with low levels of lipid droplet accumulation when compared to HFD mice. Adipocytes in HFD-DH5 mice were also significantly smaller than those on the high-fat diet alone. There were also significantly higher expression levels of PPAR-α, FABP4, and CPT1 in adipose tissues in HFD-DH5 mice, genes that are associated with lipid oxidation. This strain’s supernatant was also found to inhibit the growth of *Cronobacter sakazakii*, a pathogen with particular risk to infants, in nutrient broth [69].

*Lb. kefiri* JCM 5818 showed some ability to reduce the cytotoxic effects of *C. difficile* toxins TcdA and TcdB [62]. It also was reported to have an ability to remove cadmium, a toxic heavy metal, from its surrounding environment. This, in turn, could reduce its cytotoxic effects on cells in the surrounding environmental, such as HepG2 cells [63]. 

*Lb. kefiri* D17 displayed a number of candidate probiotic properties [10]. While it was not especially adhesive to Caco-2 cells, it showed strong cholesterol assimilation abilities. In an in vivo study, conducted in mice that had been administered 10^9^ CFU/mL of this strain daily for four weeks, TGs, TC, and LDL-C were all significantly lower in treated mice, while HDL-C was significantly higher. Additionally, liver cholesterol levels were also significantly lower compared to controls. Correspondingly, fecal cholesterol levels were significantly higher.

Finally, pre-treatment of HT-29 cells with *Lb. kefiri* IM002 significantly reduced the secretion of IL-8 caused by *S. enterica* infection [71]. 

### 2.4. Other Lactobacillus Species and Strains

#### 2.4.1. *Lactobacillus acidophilus* Strains

*Lb. acidophilus* CYC 10051 from kefir showed high-level adhesion to Caco-2 cells and was able to inhibit the growth of a wide range of bacteria in a spot agar test [46].

*Lb. acidophilus* Z1L showed high auto-aggregation ability, a desirable characteristic for a potential probiotic, while also showing high-level coaggregation with *E. coli* [75]. 

*Lb. acidophilus* LA15 from kefir showed some ability to lower TC, TG, and LDL-C plasma levels in in vivo experiments but did not increase HDL-C. Mice administered this strain also had significantly lower liver cholesterol levels combined with correspondingly higher fecal cholesterol levels [10]. 

In a randomized double-blind placebo-controlled clinical trial, patients suffering from type two diabetes were administered 600 mL/day of kefir (containing unspecified *Lb. acidophilus* and *Lactobacillus casei* strains) for eight weeks. Kefir significantly reduced levels of HbA1C compared to baseline. However, TC, TG, and HDL-C plasma levels did not change [73]. 

#### 2.4.2. *Lactobacillus paracasei* Strains

*Lb. paracasei* MRS59 was able to strongly inhibit growth of *E. coli* and *S. aureus* in a spot agar test. It also displayed significant anti-oxidant activity and went on to display significant adhesion to Caco-2 cells, displaying probiotic potential [55]. 

In milk samples inoculated with the kefir isolates *Lb. paracasei* subsp. *paracasei* CHB 2121 and *Lb. casei* NWL63, respectively, viable counts of the pathogen *Mycobacterium bovis* BCG decreased significantly, with no detectable counts recorded after 60 h of milk fermentation [53].

*Lb. paracasei* CIDCA 8339 displayed strong adhesion to Caco-2 cells [52]. It had a strong coaggregation with *S. enterica*, and strongly diminished its invasion capabilities with regard Caco-2 cells when co-incubated with this strain. Similarly, pre-treatment of Caco-2 cells with CIDCA 8339 also diminished *S. enterica’s* association and invasion to these cells. Pre-treatment also prevented the F-actin cytoskeleton disorganisation seen in infection of Caco-2 cells by the pathogen.

#### 2.4.3. *Lb. delbrueckii* Strains

*Lb. delbruekii* CYC 10047 from kefir, which exhibited strong adhesion to Caco-2 cells, strongly inhibited *Salmonella typhimurium* attachment to these cells, while also demonstrating significant inhibition of the growth of several pathogenic bacteria in a spot agar test [46].

Incubating Hep-2 cells with *Lb. delbrueckii* subsp. *lactis* (strain CIDCA 133) for one hour prior to *E. coli* infection resulted in a minor but significant effect in reducing the amount of *E. coli* adhering to Hep-2 cells [9]. It also significantly reduced the levels of cell detachment of Hep-2 cells caused by *E. coli* infection. Furthermore, it nearly abolished the F-actin disorganization and abnormal cell morphology caused by *E. coli* infection. 

*Lb. delbrueckii* ssp. *bulgaricus* B-30892 was tested for its effect against the cytotoxicity of *C. difficile* against Caco-2 cells [77]. Phase contrast microscopy displayed significantly diminished cytotoxic effects when the cell free supernatant (CFS) of strain B-30892 was co-incubated with CFS of *C. difficile* as compare to *C. difficile* alone. Immunofluorescent staining of live and dead Caco-2 cells following exposure to the CFS of these strains demonstrated that exposure of *C. difficile* killed a vast majority of the cells present, while co-incubation with strain B-30892 lead to an almost complete reverse of this trend. The CFS of this strain also significantly decreased the adhesion of *C. difficile* cells to Caco-2 cells. 

#### 2.4.4. Others

*Lactobacillus rhamnosus* ATCC 53103 and *Lb. reuteri* ATCC 53609 had a small effect in reducing the viability of *C. difficile* in vitro [76].

Furthermore, a mixture of lactic acid bacteria including a *Lb. casei* strain increased NK cell activity, including cytotoxic activity against human colorectal tumor HCT116 cells [29].

## 3. Integrative Analysis and Knowledge Gaps

A significant proportion of the strains tested and reviewed here were assessed for their ability to adhere to intestinal mucus or human cells, most commonly, but not limited to, Caco-2 cells [8,9,10,11,14,23,46,52,55,57,61]. In some cases, these assays were used as an initial screen, leading to strains that did not rate highly by this metric not being investigated further [11,52,61]. While this may be an important metric, it overlooks the possibility that bacteria may be indirectly attached to the surface of the intestine through interactions with other bacteria [84,85], as part of a consortium, which may rate highly in this metric. This is highlighted by one study, in which *Lb. kefiri* D17 exhibited a strong effect on cholesterol levels while not being particularly adhesive to Caco-2 cells [10]. It also rules out the investigation of bacteria, possibly consumed regularly as part of traditional/non-sterilized foods or harbored in other reservoirs (such as the mouth or stomach), which may have transient but regular physiological effects as they pass through the gastrointestinal tract [30]. Additionally, such a screening process could tend to select for the strains that act primarily through competitive exclusion, which could lead to an overrepresentation of these strains and this mode of action. Fortunately, this concept was not lost on all authors [8]. Furthermore, Caco-2 cells lines are particularly prone to spontaneous differentiation through culture techniques [86]. Given the explosion of the microbiome field over the past 15 years, and especially the last 5 years, it is likely that cultured Caco-2 cell lines in different institutions have significant morphological and functional differences, thus making it difficult to compare adhesion data across labs. Additionally, a number of studies have demonstrated that when Caco-2 cells are cultured, they often exhibit morphological and functional features of enterocytes associated with the small intestine [87,88], while host-microbe interactions are located mainly within the large intestine/colon. Thus, this very commonly used model may limit and skew in its representation of intestinal cell phenotype. 

### 3.1. Protection from Pathogens

Across many of the strains tested, a common characteristic reported was the ability of *Lactobacillus* strains to inhibit the growth and/or toxic effects of a wide variety of pathogenic bacteria [3,5,6,7,8,9,23,26,27,31,46,52,55,59,62,76,77]. This often occurred concurrently with *Lactobacillus* bacteria pre-treatment of host cells.

It appears that *Lb. plantarum* strains act at least in part by neutralizing the toxins released by pathogenic bacteria [3,5]. Furthermore, it appears the cell wall of these strains are essential for this “mopping up” function [7,9]. However, there is also evidence that other mechanisms may be at play, as some strains release a molecule that directly targets the pathogenic bacterial cells [27], and also prime the immune system to respond faster and stronger to pathogens [22,26].

The available evidence suggests that *Lb. kefiranofaciens* strains do not act by sequestering toxins released by pathogens [31]. More direct mechanisms of action are likely, such as the release of a bactericidal EPS [88], or possibly altering gene expression in these pathogens [15]. Modulation of the immune system to be more responsive to pathogens is also a putative mechanism [31].

*Lb. kefiri* strains appear to act, at least in part, through the properties of their cell wall, much like *Lb. plantarum* strains. Studies involving these strains have identified the importance of S-layer proteins in the mechanism of action [57], and importantly, in the sequestering of toxins [62]. 

Knowledge gaps could perhaps be narrowed by comparing *Lb. plantarum* and *Lb. kefiri* genomes and identifying common genes for S-layer proteins, which may not be present in other species such as *Lb. kefiranofaciens*, given there was no evidence for this mechanism of action (sequestration of toxins) in this species. However, given these proteins are very diverse, a high degree of variation may be expected [89]. It is possible that analysis of the sequence of various toxins may give insight into structure of the corresponding S-layer protein that targets them. Interestingly, *Lb. plantarum* and *Lb. kefiranofaciens* strains both showed some evidence of priming the immune system to pathogens, and so may share common genes or sequences that code for proteins that can interact with eukaryotic receptors. However, the specificity of such immune priming by lactobacilli is open to question, and the likelihood of such a response being abrogated by tolerization upon repeated exposure needs to be borne in mind.

### 3.2. Immunomodulation

Immunomodulation refers to the notion that certain bacteria, usually commensals or food-borne, can alter the activity of the innate immune system in such a way as to promote or inhibit responses to a subsequent challenge. Most of the work on immunomodulation has been completed with *Lb. kefiranofaciens* M1 [30,32,33,34,37], although there is a little information for strains of *Lb. plantarum* [23] and *Lb. kefiri* [68].

Treatment with the supernatant of *Lb. kefiranofaciens* strain M1 lead to significant upregulation of various pro-inflammatory cytokines in macrophages. It is proposed that this effect acts through a molecule that binds to TLR-2 and that it has a molecular weight larger than 30 kDa. It is also ruled out that the effector macromolecule is peptidoglycan, which was not detectable in the supernatant. Temperature-dependent decreases in cytokine production suggest that the protein is not heat stable or that the cells need to be living to elicit their effects (i.e., it is likely the cell wall is not involved) [37].

Later research found that a number of genes for increased immune response, inflammation, and cell adhesion were upregulated by this strain, and other genes involved in the classic complement system and the lectin-induced pathway were downregulated. Together, this fits in with the change in cytokine levels, and suggests alteration to the Th1/Th2 balance in the favor of Th1 cells [33,34], which corroborates with decreased allergic responses and lower IgE levels. Additionally, while increased Th1 activation is generally associated with an increased antimicrobial response, genes involved in the classic complement system and lectin-induced pathway were downregulated. However, these pathways are innate and not specific. Downregulation suggests these bacteria have the ability to communicate with the human immune system in such a fashion so as to promote their colonization/tolerance. This may be a feature of the putative mechanism by which certain bacteria are tolerated in early life and become part of the stable microbiome for the majority of the lifespan, especially considering the human adaptive immune system does not fully mature until 2–3 years post-birth. Considering that these are the years in which tolerance is built, bacteria that can avoid activating the innate immune system (the main component of the immune active during these years) are more likely to elicit tolerance and long-term colonization. Bacteria that are able to do this and also upregulate Th1 activity later in life would benefit from the immune system being active and more robust against *other* bacteria, once again consolidating their niche in the gut. 

This strain also acted through TLR-2 to ease symptoms of DSS-induced colitis. Interestingly, under these circumstances, *Lb. kefiranofaciens* M1 decreased pro-inflammatory cytokines, suggesting that it may depend on environmental conditions such as the type of immune response this strain may elicit from host cells [32]. However, it is perhaps more likely that this strain acts through a strengthening of the epithelial barrier through TLR-2 activation, leading to overall reduced inflammation.

It is possible the increase in pro-inflammatory cytokines associated with lactobacilli is due to priming the immune system to potential pathogens, keeping it in a state of heightened responsiveness. This is best illustrated in a study by Fuentes et al. wherein mice whose immune system was compromised had a proliferation of segmented filamentous bacteria in their gut. However, this proliferation was abolished by treatment with *Lb. plantarum* C4 [24]. This strain also increased levels of splenocytes in these mice [23]. This theory is further supported by increased TLR immune response to TLR agonists in mice treated with M1 [30].

Immunomodulation properties are also, in some cases, anti-carcinogenic. Several strains have been associated with such anti-carcinogenic properties [29,67,68]. Unspecified *Lb. plantarum* and *Lb. casei* strains were found to upregulate cytotoxicity of NK cells against various cancer cell lines [29]. *Lb. kefiri* P-IF was also found to increase levels of cell death amongst gastric cancer cells [67]. The increase of co-stimulatory markers detected on DCs treated with this strain may be a putative mechanism of action, making detection of cancer cells more likely [68]. Additionally, these DCs, when incubated with CD8^+^T cells, upregulated CD107a and granzyme-B expression, markers of cytotoxic T cell activity.

### 3.3. Reduction of Cholesterol Levels

Multiple studies demonstrated the ability of lactobacilli to either reduce levels of cholesterol circulating in the plasma, levels of cholesterol in the liver, and/or increase levels excreted, mostly in response to treatment with *Lb. plantarum* strains [10,11,14,20], but also one *Lb. kefiri* strain [70]. This reinforced the theory of the significant role the microbiota plays in cholesterol removal, although some of these effects are indirect, mediated by microbial modulation of bile metabolism, which impacts on liver metabolism [90]. Furthermore, it appears that treatment with these bacteria leads to significantly lowered levels of LDL cholesterol, the type more so associated with heart disease. In some studies, HDL cholesterol, traditionally seen as the “good” cholesterol, was unaltered, while LDL was significantly reduced [10,11,14,70]. Lactobacilli also significantly reduced levels of TG in many of these studies. This has been further correlated with a significant decrease in levels of atherosclerotic lesions found in the aorta of rabbits fed a high-cholesterol diet supplemented with lactobacilli [44]. However, the mechanisms behind these common characteristics was not investigated. Thus, functional genomic studies should be undertaken of strains with and without these characteristics.

### 3.4. Antioxidative Effects

Several studies reported that *Lb. plantarum* had modest antioxidative effects, appearing to upregulate activity of several enzymes catalyizng free radicals [18,19]. It is thought that these effects may be due, at least in part, to the high scavenging ability of the EPS of these bacteria [13].

## 4. Conclusions

Kefir has been associated with health benefits for decades. Now, there is clear scientific evidence emerging as to which microbes are responsible for individual effects. Lactobacilli are particularly characterized in this regard, being associated with protection from pathogenic bacteria, modulation of the immune system to potentially reduced risk of allergies and cancer, reduction of radical oxidative species and cholesterol levels, and potentially benefiting in diabetes. However, there are major deficiencies in understanding of the mechanisms behind these reported effects, and how many of these reported benefits translate from in vitro and pre-clinical models to human consumers. A far greater level of knowledge into the mechanisms, which may underpin these claimed benefits, is required. Comparative genomic studies of phenotypically well characterized kefir strains would be of additional benefit in this context, and relatively easy to carry out considering the explosion of high-throughput sequencing technology in recent years. Identification of key genes involved in health benefits would be a major step forward in exploiting kefir lactobacilli. While it is acknowledged that the study of individual strains outside of their natural microbial consortia imposes certain significant limitations, it seems pertinent from the literature that the significant variation in effect, even between strains of the same species, warrants further study. Certainly, beneficial effects appear synergistic when considering multi-species models of kefir compared to single strain or lower number models. However, single strain models can help identify the primary source of such effects, upon which other species may contribute to in a secondary, albeit significant, fashion. Furthermore, there would be great difficulty in attempting to tease out individual effects with full kefir on an in vivo scale. Additionally, considering the fact that the construction of an artificial kefir grain is a significant goal of the dairy industry, the knowledge afforded by such studies may one day become particularly relevant regarding the choice of strains to be included in such grains, perhaps eventually allowing relatively targeted consortia for particular functional properties. Given the mooted paradigm shift towards personalized medicine, such studies are likely to grow in importance.

## Figures and Tables

**Table 1:** List of *Lactobacillus* species and strains found in milk kefir.

*Lactobacillus* spp.	Strain and Reference	*Lactobacillus* Phylogenetic Group ^1^	Type Strain	Genome Size (Mb)
**Lb. plantarum**	-Lb. plantarum CIDCA 83114 [3,4,5,6,7,8,9] -Lb. plantarum CIDCA 8336 [3,8] -Lb. plantarum B23 [10] -Lb. plantarum Lp27 [11] -Lb. plantarum YW11 [12,13] -Lb. plantarum Lp09 [14] -Lb. plantarum Lp45 [14] -Lb. plantarum ATCC 10012 [15] -Lb. plantarum K25 [16] -Lb. plantarum MA2 [17,18,19,20] -Lb. plantarum CIDCA 8327 [8,21] -Lb. plantarum C4 [22,23,24,25,26,27,28] -Unspecified [29] -Lb. plantarum CIDCA 8337 [8]	Lb. plantarum	Lb. plantarum subsp. plantarum CGMCC 1.2437	3.2
**Lb. kefiranofaciens**	-Lb. kefiranofaciens M1 [30,31,32,33,34,35,36,37] -Lb. kefiranofaciens ssp. Kefirgranum [38] -Lb. kefiranofaciens HL1 [39] -Unspecified [40,41,42,43,44,45] -Lb. kefiranofaciens CYC 10058 [46] -Lb. kefiranofaciens DD2 [15] -Lb kefiranofaciens JCM 6985 [47] -Lb. kefiranofaciens DN1 [48] -Lb. kefiranofaciens KCTC 5075 [49] -Lb. kefiranofaciens ZW3 [50] -Lb. kefiranofaciens ATCC 43761 [51]	Lb. delbrueckii	Lb. kefiranofaciens subsp. kefiranofaciens DSM 5016	2.6
**Lb. paracasei**	-Lb. paracasei CIDCA 8339 [52] -Lb. paracasei CHB 2121 [53] -Lb. paracasei CIDCA 8339 [54] -Lb. paracasei MRS59 [55] -Lb. paracasei SP3 [56]	Lb. casei	Lb. paracasei subsp. paracasei DSM 5622	2.9
**Lb. kefiri**	-Lb. kefiri CIDCA 8348 [3,4,5,6,7,57,58,59,60,61,62,63,64] -Lb. kefiri CIDCA 83111 [59,62] -Lb. kefiri CIDCA 83102 [52] -Unspecified [42,49,65,66] -Lb. kefiri LMG 9480 [38] -Lb. kefiri P-IF [67,68] -Lb. kefiri HL2 [39] -Lb. kefiri DH5 [69,70] -Lb. kefiri CIDCA 83115 [61] -Lb. kefir JCM 5818 [62,63,64] -Lb. kefiri D17 [10] -Lb. kefir IM002 [71]	Lb. buchneri	Lb. kefiri DSM 20587	2.2
**Lb. parakefiri**	-Lb. parakefiri KP91 [38]	Lb. buchneri	Lb. parakefiri DSM 10551	2.5
**Lb. acidophilus**	-Lb. acidophilus LA15 [10] -Lb. acidophilus B2-2 [72] -Unspecified [73,74] -Lb. acidophilus CYC 10051 [46] -Lb. acidophilus Z1L [75]	Lb. delbrueckii	Lb. acidophilus DSM 20079	2.0
**Lb. casei**	-Unspecified [29,73] -Lb. casei NWL63 [53]	Lb. casei	Lb. casei DSM 20011	2.8
**Lb. sunkii**	-Unspecified [66]	Lb. buchneri	Lb. sunkii DSM 19904	2.7
**Lb. johnsonii**	-Lb. johnsonii JCM 1022 [15]	Lb. delbrueckii	Lb. johnsonii ATCC 33200	1.8
**Lb. rhamnosus**	Lb. rhamnosus ATCC 53103 [76]	Lb. casei	Lb. rhamnosus DSM 20021	2.9
**Lb. kefiranofaciens subsp. kefirgranum**	-Lb. kefirgranum KCTC 5086 [49]	Lb. delbrueckii	Lb. kefiranofaciens subsp. kefirgranum DSM 10550	2.1
**Lb. brevis**	-Unspecified [75]	Lb. casei	Lb. brevis DSM 20054	2.5
**Lb. delbrueckii**	-Lb. delbruekii CYC 10047 [46] -Lb. delbrueckii subsp. Lactis CIDCA 133 [9] -Lb. delbrueckii ssp. bulgaricus B-30892 [77]	Lb. delbrueckii	Lb. delbrueckii subsp. delbrueckii DSM 20074	2.0
**Lb. helveticus**	-Lb. helveticus Z5L [75]	Lb. delbrueckii	Lb. helveticus CGMCC 1.1877	2.0
**Lb. bulgaricus**	-Unspecified [75]	Lb. delbrueckii	Lb. delbrueckii subsp. bulgaricus DSM 20081	1.8
**Lb. fermentum**	-Lb. fermentum ME-3 [78]	Lb. reuteri	Lb. fermentum DSM 20055	1.9
**Lb. reuteri**	-Lb. reuteri ATCC 53609 [76]	Lb. reuteri	Lb. reuteri DSM 20016	2.0

^1^ According to [2] Salvetti, E.; M. B. Harris, H.; Felis, G.; W. O’Toole, P. *Comparative genomics reveals robust phylogroups in the genus lactobacillus as the basis for reclassification*. 2018; Vol. 84, p AEM.00993-00918.
